# METHODOLOGICAL APPROACHES TO GERONTOLOGICAL CANCER RESEARCH

**DOI:** 10.1093/geroni/igac059.586

**Published:** 2022-12-20

**Authors:** Sean Halpin

## Abstract

The wide range of gerontological cancer research necessitates a variety of methodological approaches. In our symposium, we bring together researchers who represent varied approaches to studying multiple cancer types—with a focus on demonstrating how to apply different methods. First, Ye, will discuss the use of a unique cross-sequential design to facilitate comparison between health change in long-term older cancer survivors and demographically-matched older adults with no history of cancer. Zanwar, will present disparities in cancer screening using secondary nationally representative complex survey data, provide examples of survey data that can be utilized in aging and cancer prevention and control research, and present challenges and opportunities for using survey data. Von Ah, will discuss research methods pertaining to a series of non-pharmacological clinical trials and offer insight to reducing barriers and improving acceptability to technology-based intervention programs in older breast cancer survivors. Next, Castaneda will present quantitative (i.e., group trajectory modeling, conditional Poisson regression) and qualitative approaches to understand the role of monoclonal gammopathy of undetermined significance in healthcare utilization and progression to multiple myeloma. Last, Halpin will discuss the use of naturally occurring data such as participant observation and audio recordings to evaluate education for patients with multiple myeloma preparing for autologous stem cell transplant. Understanding how a variety of methodological approaches are applied to gerontological cancer research will help facilitate a broader understanding of the tools available for these studies.

